# Modern Sociology

**Published:** 1902-01-11

**Authors:** 


### Modern Sociology.

### WOMEN'S RESTAURANTS (BY OUR SPECIAL COMMISSIONER).

### I.—The Problem.

The problems of our large towns grow year by year more pressing and more complex, and not the least pressing problem awaiting solution is the food of the people. It will be enough, in these papers, if we confine ourselves to the consideration of the wage-earning woman, for though the working man's meals may not be as good and nourishing as they might be, owing to ignorance and bad management at home, yet as far as public restaurants are concerned, he is incalculably better off than the women workers.

Mr. Sidney Webb has somewhere said that women will never adequately compete with men until they learn to eat more. The man knows by experience that he cannot do his work without a good dinner, and the public caterer is able consequently to give him a good dinner at a reasonable price, for there is certainty of custom and a sure profit on large quantities. But how are women to learn to eat more when they have not the money to spend on "more"? The whole question of women's wages comes in here, and so long as the working girl continues to be satisfied with a much lower rate of payment than her brother for an equal amount of work, so long as she refuses to combine to improve her social status, just so long will her wages continue to be low and her spending capacity small.

There are other considerations. "Women put their money on their backs instead of in their stomachs" is one way of expressing the assertion that too large a proportion of a girl's earnings goes in dress. Again, their work is largely sedentary, and their health consequently uncertain; anæmia is common, and the appetite is frequently dainty.

The large plate piled with meat and vegetables which the carter relishes after a morning's work in the open air, is enough to sicken the girl who has been plying the needle in the workroom, while he has been smacking his whip along a country road. This is one reason why, out of the 900 or 1,000 women and girls who dine daily at the Alexandra Trust Restaurant in the City Road, only perhaps two dozen have the 5½d. meat dinner of soup, joint and two vegetables and pudding. By far the larger number dine off half-haddocks at 1½d., tinned salmon at 1d., a couple of sardines, scones and pastry, or tea.

The question of wages is, however, the crux of the matter. Cheap as the above-mentioned meat meals are, they are not cheap enough for the girl earning 6s. or 7s. a week, for out of this how is she to pay 5½d. a day for one meal, even though it is the chief meal in the day? "Such girls," says Miss Clara James, whose long experience of the London working-girl entitles her to be heard, "seldom think of going into a restaurant for food. This is what they eat:—Cold meat and bread on Monday; fish and bread or cheese and pickles on Tuesday, Wednesday and Thursday; Friday is generally hard up day, when they either borrow a penny or go for a walk in the dinner-hour, to look in the shops at the well-to-do feeding. Out of 6s. or 8s. per week a girl, even if she lives at home, would not think of spending so much as 6d. on one meal, for even out of that small wage employers want respectable girls, don't forget that. The cause of the evil lies in the selfishness of the exploiter of labour." And she added in reply to our inquiries, "If a girl is to have a holiday at all in the summer, she nearly always saves the money out of what should be her food-money. How is she to get her holiday else, unless she accepts charity?"

Miss Eves, of the Maurice Hostel (Women's Branch), Hoxton, throws another ray of light on this difficult question. "Whatever you give the working girl for dinner," she said, "you have got to convince her that it is not only better, but cheaper, than she could get for herself." A woman has a way of going to the root of things, that is to say, which, owing to his different training and experience, a man lacks; and an interesting illustration of this fact occurred while we were prosecuting our inquiries on the subject of women's restaurants. Into a public tea-room there came a lady and gentleman; the latter ordered a cup of cocoa, and was somewhat annoyed to find that it was 3d. instead of 2d. "I wish I hadn't had it," was his comment as he drank his money's worth; but his wife fell to musing. Calling the waitress, she inquired what cocoa it was, and being told, remarked that she used that cocoa at home, and that one cup could not possibly cost 3d. to make. She had mentally gone into the process of making, had measured the teaspoonfuls she would have used, and the whole thing appeared to her a preposterous over-charge. But she had only half done the mental inquiry; and a little business training would have told her that out of the 3d. on that cup of cocoa, there was not only the actual price of the cocoa and milk to cover, but there was the cost of fuel for cooking it, crockery to put it in, rent of the restaurant, wages of the cook and waitress, breakages, and a thousand other considerations which somehow or other had to be met, and a proportion of the burden of which fell upon that particular cup of cocoa.

The girl you cater for must be convinced that she is getting her money's worth, for she is quick-witted enough to know what it would cost her to fry a bit of fish at home, and so your price must be low and the quality good, or she will not eat at your table.

The letter sent by Miss O'Kell, Sanitary Inspector for Marylebone, to the papers a few weeks ago, draws attention to the need that exists for cheap restaurants—especially in the West End. "In the course of my daily duties," she says, "which bring me into close contact with the working girl of the West End, the pressing need of special restaurants for women and girls is constantly presenting itself. It is obvious that for the rapidly growing girl, whose strength is further taxed by sedentary occupations in close rooms for long consecutive hours, abundance of nourishing food is essential. In the workrooms of the dressmakers and milliners of the West End there are thousands of young girls employed, from the child-apprentice earning a weekly half-crown for 'pocket-money,' to the finished assistant who receives on an average 15s. to 18s. per week. These are absent from their distant homes for periods of 12 hours and longer, during which time a nourishing meal is unobtainable. The prices of the West End restaurants are, needless to remark, beyond the reach of these workers. At the mid-day break the only alternative is a lunch at the tea-shop, where the few pence are spent in worse than useless fare, or the consumption, in the workroom, of the unappetising dry sandwich. Is it to be wondered at that so many of our girls break down at the onset; that many more struggle on, wearily battling with that arch enemy to young womanhood, anæmia, which is the forerunner of even worse ills?"

The question is, is it possible to provide such restaurants without resorting to charity?

